# Multi-Sensor Soil Probe and Machine Learning Modeling for Predicting Soil Properties

**DOI:** 10.3390/s24216855

**Published:** 2024-10-25

**Authors:** Sabine Grunwald, Mohammad Omar Faruk Murad, Stephen Farrington, Woody Wallace, Daniel Rooney

**Affiliations:** 1 Pedometrics, Landscape Analysis & GIS Laboratory, Soil, Water, and Ecosystem Sciences Department, University of Florida, 2181 McCarty Hall, P.O. Box 110290, Gainesville, FL 32611, USA; sabgru@ufl.edu; 2 Department of Biological Systems Engineering, Institute of Agriculture and Natural Resources, College of Engineering, University of Nebraska at Lincoln, 206 LW Chase Hall, P.O. Box 830726, Lincoln, NE 68583, USA; omurad2@unl.edu; 3 LandScan, LLC, 423 L Street, Suite D, Davis, CA 95616, USA; farrington@landscan.ai (S.F.); wallace@landscan.ai (W.W.)

**Keywords:** digital twin, digital soil mapping, soil sensors, multi-sensor system, digital soil core, machine learning, artificial intelligence, soil properties, scale

## Abstract

We present a data-driven, in situ proximal multi-sensor digital soil mapping approach to develop digital twins for multiple agricultural fields. A novel Digital Soil Core^TM^ (DSC) Probe was engineered that contains seven sensors, each of a distinct modality, including sleeve friction, tip force, dielectric permittivity, electrical resistivity, soil imagery, acoustics, and visible and near-infrared spectroscopy. The DSC System integrates the DSC Probe, DSC software (v2023.10), and deployment equipment components to sense soil characteristics at a high vertical spatial resolution (mm scale) along in situ soil profiles up to a depth of 120 cm in about 60 s. The DSC Probe in situ proximal data are harmonized into a data cube providing vertical high-density knowledge associated with physical–chemical–biological soil conditions. In contrast, conventional ex situ soil samples derived from soil cores, soil pits, or surface samples analyzed using laboratory and other methods are bound by a substantially coarser spatial resolution and multiple compounding errors. Our objective was to investigate the effects of the mismatched scale between high-resolution in situ proximal sensor data and coarser-resolution ex situ soil laboratory measurements to develop soil prediction models. Our study was conducted in central California soil in almond orchards. We collected DSC sensor data and spatially co-located soil cores that were sliced into narrow layers for laboratory-based soil measurements. Partial Least Squares Regression (PLSR) cross-validation was used to compare the results of testing four data integration methods. Method A reduced the high-resolution sensor data to discrete values paired with layer-based soil laboratory measurements. Method B used stochastic distributions of sensor data paired with layer-based soil laboratory measurements. Method C allocated the same soil analytical data to each one of the high-resolution multi-sensor data within a soil layer. Method D linked the high-density multi-sensor soil data directly to crop responses (crop performance and behavior metrics), bypassing costly laboratory soil analysis. Overall, the soil models derived from Method C outperformed Methods A and B. Soil predictions derived using Method D were the most cost-effective for directly assessing soil–crop relationships, making this method well suited for industrial-scale precision agriculture applications.

## 1. Introduction

**Figure 1:**
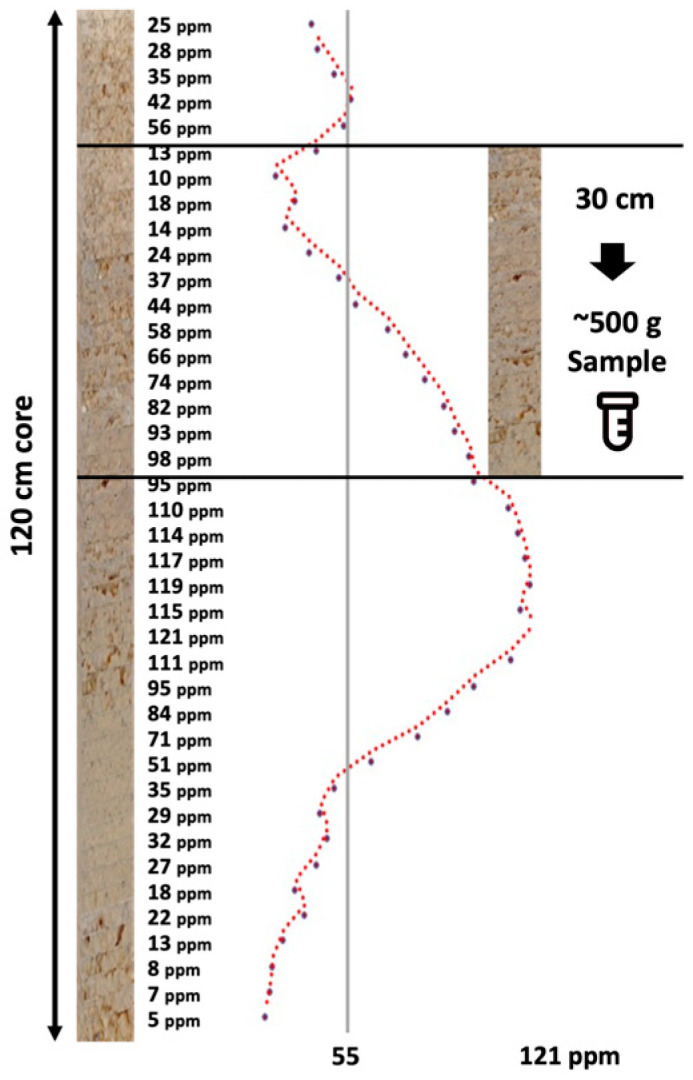
Idealized hypothetical DSC System soil data profile showing a soil property (e.g., soil potassium in ppm) derived from fine-resolution sensor measurements and a conventional coarse ex situ soil sample (~500 g soil) collected within a soil layer with a 30 cm depth.

The need for cost-effective, rapid, deep, and comprehensive soil health characterization in support of climate-smart agricultural management, soil carbon accounting, precision agriculture applications, and digital twins in smart farming is profound [1,2,3]. Traditional approaches to characterize soils are laborious, entailing the ex situ collection of soil samples in individual horizons/layers or soil coring, soil analytics in the laboratory, and digital soil mapping and modeling. In situ proximal sensing in the near surface dates to the late 1990s and early 2000s with Ben-Dor et al. [4], who reported the first instrument of its nature, a soil penetrometer [5], that was later coupled to a window regulating mechanism that collected reflected light, enabling one to view the color and structure of the soil profile [6]. Poggio et al. [7] conducted a laboratory-based evaluation of the optical performance of a soil penetrometer that included visible and near-infrared (VisNIR) optics, which acknowledged the contributions of Rooney to the design. Recently, the advent of proximal soil sensor technology and artificial intelligence (AI) soil predictive modeling has excelled to quantify soil health properties, especially soil organic carbon [8]. Soil measurements made in the laboratory under controlled conditions are still considered the “gold” standard in terms of the accuracy and precision of measurements, with soil sensors calibrated and validated against these standards. However, the mismatch of the spatial scale and sample support of laboratory and in situ field sensors is stark and has not been sufficiently addressed in research investigations. The sample sizes required to support conventional laboratory analysis often substantially exceed the spatial scale within which soil properties vary. Proximal sensors are in close proximity to soil samples, with the potential to continuously characterize the variability of soils along soil profiles [9], while conventional extracted soil samples used for laboratory-based soil analytics are low in volume, mass, and vertical resolution. Figure 1 demonstrates the relationship between in situ proximal sensing and soil sampling for the measurement of soil potassium. The proximal sensor delineates the profile at a sub-centimeter scale, which represents how potassium is distributed in nature. However, the laboratory requires 500 g of soil sample, which equates to about a 30 cm section of a standard core, to run a full laboratory testing panel. Of this 500 g, approximately 50–75 g is utilized for nutrient testing. The result from the lab shows one value (55 ppm), while the range detected by the proximal sensor varies from 10 ppm to 98 ppm. Which is more likely to be a true representation of how potassium is distributed in the soil profile? How to compare the two? Which is the “gold” standard?

Another shortcoming of ex situ soil sampling is that the in situ co-relation between soil properties and attributes is disrupted during extraction, which further degrades the utility of soil information derived from conventional methods and obfuscates the intent of the survey. For example, when mapping soil and water properties for irrigation management at the field scale, understanding the in situ relationship between the grain size and packing, structure, density, microbial gums, and the depth and thickness of soil horizons is critical. By removing a soil sample and performing a laboratory soil texture test, all of the corresponding contextual soil information is detached, thus degrading the value of the soil texture data as a predictor of the water-holding capacity. This is typical of each laboratory testing procedure, whether physical, chemical, or biological. A precise holistic understanding of soil–crop relationships is best informed by in situ soil testing conducted where the roots interact with the soil properties.

In addition to the issues with vertical resolution and the loss of sample context, conventional methods are also at a disadvantage with respect to a lower spatial resolution. In practice, where what is learned during research is intended to be put to practical use in agriculture, it is not realistic to obtain and test enough ex situ soil samples to create an accurate map with the spatial resolution needed to operate currently available variable-rate nutrient and soil amendment applicators and irrigation technologies. The result is that ex situ soil information is interpolated and extrapolated and then digitized in ways that are not recognized or challenged by end-users. The resulting ex situ soil data are highly subjective and lack spatial and information resolution, and are not suitable for advanced analytics enabled by a digital twin.

Some of the understudied research questions include (1) whether the approach to ground-truth in situ field soil sensor data with manually extracted coarser-scale ex situ soil samples undergoing laboratory soil analysis realizes the full potential of the in situ proximal sensing of soil properties, and (2) which scaling function performs best to link high-resolution in situ soil sensor data and coarser resolution ex situ laboratory analytic data.

The most widely studied proximal soil sensors are visible–near-infrared (VNIR) and mid-infrared (MIR) spectral instruments, which have been used to develop soil spectral libraries at global [10,11], regional [12,13], and national scales, for example, in the U.S. [14,15], Brazil [16], China [17], and Switzerland [18]. The ability to predict soil organic carbon (SOC) using machine learning (ML) with large-scale spectral libraries in the U.S. has shown excellent performance using independent validation data. For example, SOC predictions from VNIR spectra and random forest (RF) modeling achieved a Coefficient of Determination (R^2^) of 0.95, a Ratio of Performance to Inter-Quartile (RPIQ) of 0.81 [14], and an R^2^ of 0.96 and an RPIQ of 5.18 [19] using rigorous validation assessment for soils in the conterminous U.S. Similarly compelling results in the validation mode were achieved for modeling the SOC in the U.S. using VNIR spectra and Convolutional Neural Networks (CNN-1), with an R^2^ of 0.83 and RPIQ of 0.81, and even better results using MIR spectra and CNN-1, with an R^2^ of 0.98 and RPIQ of 2.37 [15]. Other physical and chemical soil properties, such as the macro- and micro-nutrients, soil texture, cation exchange capacity (CEC), and pH have been predicted widely from diffuse reflectance spectral data [14,20,21,22,23]. In particular, MIR spectral data have fingerprinting capabilities for soil characteristics and the elemental content, while VNIR relies on the overtones of chemical bonds in the spectra (e.g., C–O, C–H, N–H, and O–H) [12].

Bulk density (BD) cannot be directly inferred from spectral reflectance data because it relies on associations with other soil properties such as the soil texture and SOC. For example, the BD (measured using clod-only, core-only, and combined clod and core methods) was predicted using Partial Least Squares Regression (PLSR), Cubist, memory-based learner (MBL), and RF from MIR data, with an R^2^ in validation mode ranging between 0.64 (PLSR) and 0.81 (MBL) [24]. Davari et al. [25] found that both the soil BD (R^2^ = 0.35) and soil porosity (R^2^ = 0.16) were poorly predicted using only VNIR spectra, suggesting that other sensors, such as penetrometers that measure tip and sleeve stress, are needed to improve the inference capabilities [26,27]. The Soil Condition Analysis System (SCANS) integrates an ex situ soil core scanning system with multiple sensors, including a γ-ray attenuation densitometer to measure the BD, digital cameras for soil imaging, and a VNIR spectrometer [28].

The advantages of spectral soil prediction modeling include that VNIR provides high sample throughput through the rapid scanning of samples compared to conventional soil analytics [29,30]. Hyperspectral soil data show significantly higher information content than traditional laboratory soil analytics. Proximal soil sensing is non-destructive and produces no hazardous materials. Another advantage is that, once large spectral libraries have been built, they can be reused and improved (e.g., applying novel ML algorithms) over time until they reach model saturation. Review articles of proximal soil sensing technology unequivocally converge in view that the proximal soil health sensing of individual soil samples is a mature analytical technique if performed under controlled laboratory conditions using the sieving, grinding, and drying (MIR), and sieving and drying (VNIR), of soil samples [31,32,33,34]. Sieving and drying operations are employed to produce comparability among laboratory scanned spectra because soil reflectance spectra are also affected by the particle size [35,36,37,38,39] and surface roughness [40,41,42], both of which relate to the soil texture.

The emergence of field-based soil spectroscopy using portable or mounted instruments has marked a shift from laboratory settings to in situ field sensing [43]. Some field studies showed significant differences between controlled laboratory- and field-based VNIR applications due to spatially variable environmental conditions. For example, the study by Hedley et al. [44] used a portable spectroradiometer to predict the topsoil SOC from field-moist spectra, with a low R^2^ = 0.39 and Ratio Performance Deviation (RPD) = 1.28, compared to air-dry spectra, with an R^2^ = 0.80 and RPD = 2.25, which showed significant differences due to the effects of soil moisture. The effects of soil moisture on soil spectral modeling have long been known in the spectral soil community [41,45,46]. According to Seidel et al.’s empirical data [47] (2022), soil moisture effects are more significant in MIR than VNIR applications. Methods such as external parameter orthogonalization (EPO), direct standardization (DS), global moisture modeling (GMM), slope-bias correction (SB), and selective wavelength modeling (SWM) have been suggested to address the application of VNIR under field conditions with varying soil moisture contents [19]. In their study, dry samples were rewetted with different soil moisture contents, demonstrating that EPO, DS, and GMM account satisfactorily for the effect of moisture in soil spectra. These three methods improved the prediction of the SOC substantially, with an increase in the R^2^ from almost 0 for no correction to over 0.5 and an RPIQ from 0.38 to over 1.7. These findings suggest that the effect of moisture on the VNIR modeling of the SOC and other soil properties is removable through post-process corrections applied to the spectral data. Knadel et al. [48] provided a comprehensive review of mathematical techniques to remove the moisture effects from the VNIR spectra. However, such approaches are computationally expensive if applied to spectral field data. Data-driven ML methods offer alternatives to the removal of soil moisture effects from spectral data by explicitly incorporating moisture data along with spectral and/or other sensor data into soil prediction models.

One such study was presented by Zhou et al. [23] (2024), who analyzed loess soil samples to investigate how changes in the soil moisture content impact predictions from VNIR spectra. Various supervised learning and latent variable methods (PLSR, RF, and Support Vector Machines) were tested with the first derivative-Genetic algorithm (GA)–RF method, demonstrating successful predictions of the soil moisture, with an R^2^ of 0.99 and Relative Prediction Deviation (RPD) of 16.2. Similarly, Lobell and Asner [49] quantified the strong influence of moisture on spectral reflectance and absorption features. Tan et al. [50] critiqued that many studies using soil spectroscopy focused on dried soil samples in the laboratory under controlled conditions, while techniques to remove the soil moisture effects from VNIR spectra are time-consuming and counter-productive in the field. In Tan et al.’s [50] empirical study, soil moisture effects were successfully eliminated from VNIR spectra to model the soil organic matter (SOM) using Principal Component Analysis (PCA)–RF coupled with the continuous wavelet transform (CWT). They found that wavelengths at about 580 nm, 820 nm, and especially the narrow region around 1400 nm are highly correlated regions to the SOM using wet soil samples. Validation results to predict the SOM from wet samples based on PCA-RF (R^2^ = 0.84 and RPD = 2.53) and dry samples (R^2^ = 0.86 and RPD = 2.68) were statistically equivalent [50]. These results suggest that in situ proximal sensing under varying soil moisture conditions combined with ML can achieve similarly good soil predictions as those derived from controlled conditions in the laboratory.

ML algorithms have been widely applied in the emerging field of predictive soil modeling using portable spectroradiometers that characterize soils under field conditions. Portable VNIR and MIR approaches have shown promising results using the PLSR modeling of soil carbon and other soil health properties when compared to lab-based diffuse reflectance spectral measurements [27,51,52,53,54,55,56,57]. According to Hutengs et al. [55], portable VNIR and MIR instruments provided accurate models of various soil physicochemical properties (an R^2^ between 0.72 and 0.99) that showed some influence by the soil moisture state (dry vs. field-moist). Validation models for the SOC achieved an R^2^ of 0.82 (dried, VNIR), 0.88 (dried, MIR), 0.57 (field-moist, VNIR), and 0.72 (field-moist, MIR). In the study presented by Semella et al. [56], SOC predictions from both VNIR and MIR spectra collected with portable spectroradiometers were equally highly reproducible on average, with a slightly higher robustness in the MIR. The results showed that the contributions of spectral variation (∆RMSE < 0.4 g kg^−1^; RMSE: Root Mean Square Error) and the reference SOC uncertainty (∆RMSE < 0.3 g kg^−1^) to spectral modeling errors were small compared to the difference between the VNIR and MIR spectral ranges (∆RMSE~1.4 g kg^−1^ in favor of MIR). Studies with handheld single-sensor instruments, such as the ASD Labspec 2500 [51], Quick Carbon Reflectometer [58], Agilent 4300 handheld FTIR [53], AgriSpec [57] (Sharififar et al., 2019), NeoSpectra [27,57,59], NanoQuest [60], and Hamamatsu C12880MA [27], demonstrate the capabilities to sense the SOC and other soil properties, though with variable results based on the sensors’ capabilities. One major disadvantage is that these portable instruments require soil samples to be extracted to be sensed in the field and they do not allow in situ continuous sensing along soil profiles. These kinds of quasi-in situ VNIR sensing systems require soil cores to be first extracted and then scanned using a field spectroradiometer [44]. Tractor- or truck-mounted sensors cover the full VNIR spectral range, but due to the vehicle movement during data collection, often the uncertainty in soil predictions can be substantial [27,61]. Soil sensors that do not possess in situ penetration capabilities severely limit the characterization of soil spatial variability, especially in crops with extensive rooting systems.

A comprehensive characterization of a suite of soil health and other profile properties and attributes in agriculture applicable to a wide variety of cropping systems (e.g., specialty crops, row crops, and different crop species) calls for multiple sensors to be used in combination that are fully integrated into a soil sensing system. Often, single-sensor instruments are applied separately to map specific soil characteristics, and then the data are fused later during the data processing and modeling phase [62]. For example, individual sensors, such as apparent electrical conductivity (ECa) to map the soil salinity [63], portable X-ray fluorescence (pXRF) spectrometry for elemental and soil fertility characterization [64], and high-capacity tensiometers, microwave-based approaches, and others for soil moisture sensing, provide specialized applications. Schmidinger et al. [65] compared the model performance of six independent in situ proximal soil sensors, one remote sensor (Sentinel-2), and all of the sensor data fused together to predict the SOM, phosphorus (P), magnesium (Mg), potassium (K), moisture, and pH with multiple ML algorithms. Five out of six soil properties achieved an R^2^ ≥ 0.80, often with various combinations of individual sensors, while, unsurprisingly, the improvement derived from fusing an increasing number of sensors was subject to diminishing returns. Similar testing of soil model performance to assess the effectivity of multiple single-sensor combinations (less than a max. of four) and fused sensor data were presented by Chen et al. [66] (2021), Tavares et al. [67], and Xu et al. [68]. Vasques et al. [69] applied multiple sensors (the ECa, apparent magnetic susceptibility meter, gamma-ray spectrometer, water content reflectometer, cone penetrometer, and pXRF) in a pasture field and found that multiple soil sensor data fused together improved the soil predictions for all soil properties relative to single sensors. The pXRF data produced the best predictions for the SOC, clay content, and BD, standing out as the best single sensor for soil property prediction, whereas the other sensors combined outperformed the pXRF sensor for the sum of bases, CEC, and soil volumetric moisture based on independent validation. These findings suggest that different combinations of sensors are needed to provide inference on a variety of soil physical and chemical properties.

Although the integration of multiple sensors into a mobile platform has sparked profound interest in the agronomic and soil science communities, fully integrated systems are rare and typically limited to a few sensors. An early attempt at a multi-sensor system for soil physical properties was presented by Yurui et al. [70]. The Veris P4000 multi-sensor instrument can collect VNIR spectra, ECa, and cone index (CI) penetrometer readings up to 1 m depth. In Pei’s study in two fields in central Missouri, U.S., the Veris P4000 achieved modest results in cross-validation mode, with average R^2^ values across all soil properties (the SOC, total nitrogen—TN, soil texture, CEC, Ca, Mg, K, and pH) for the PLSR, neural network (NN), Regression Trees (RT), and RF of 0.59, 0.46, 0.39, and 0.45, respectively. While a few properties achieved promising results with the PLSR (e.g., an R^2^ of 0.81 for the SOC), some properties showed a weak model fit (an R^2^ of 0.37 for the sand content). A multi-sensor robotic platform with a modular sensing box that includes VNIR, a thermal camera, two visual cameras forming a stereo couple, and an Inertial Measurement Unit (IMU) that provides navigational data mounted on an autonomous vehicle to generate 3D ground maps for precision agriculture applications was described by Milella et al. [71]. Other multi-sensor soil systems are static and intended for real-time sensing at only one specific location. For example, a buried soil probe containing electrochemical sensors in a hygroscopic membrane to monitor soil nutrient concentrations in real time was combined with an air probe that collects information regarding environmental conditions and gaseous emissions (esp. NH_3_, N_2_O, and CH_4_) just above the ground, and smart data loggers connecting to the Internet of Things (IoT) cloud [72] (Balan et al., 2020). Such static soil sensor systems lack the mobility to collect data across farms and cropping systems to optimize climate-smart and practical agricultural management.

In this paper, we present research using an in situ proximal soil sensing system designed and deployed by LandScan, LLC (Davis, CA, USA), that includes a multi-sensor probe, software, and equipment to deploy (DSC System). The research objectives include investigating the capabilities of the DSC System to predict various soil health and management-related properties, as well as directly predicting crop metrics without the use of ex situ soil samples and laboratory analytics, and the effects of the mismatched scale between high-resolution in situ proximal sensor data and coarser-resolution ex situ soil laboratory measurements to develop soil and plant prediction models used to create a digital twin. We critically discuss the limitations of the contemporary paradigm to ground-truth soil sensor data with laboratory-based ex situ soil measurements and present an alternative method that focuses on measured soil–crop responses.

### Study Area

**Figure 2:**
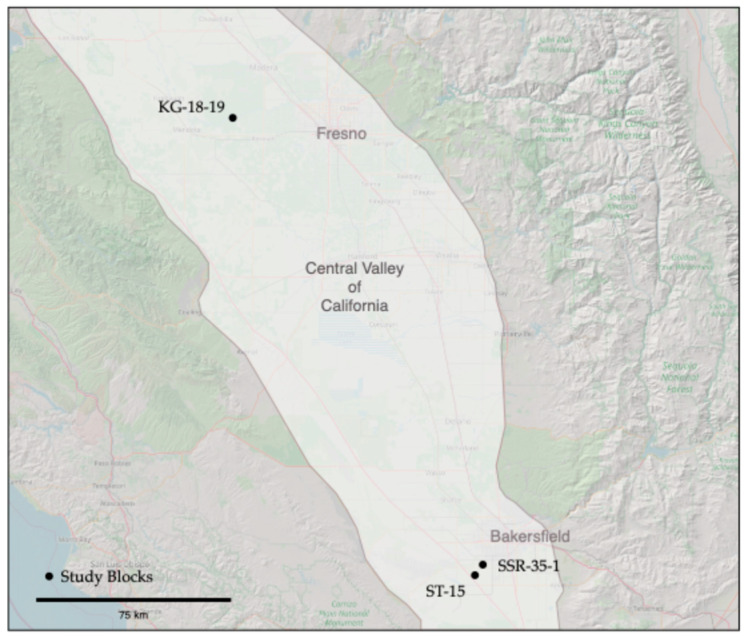
Map showing the location of the study blocks relative to the Central Valley of California, U.S., and the closest cities.

Data collection for this study was conducted across three almond management blocks located on commercial ranches in central California (Figure 2). The first ranch is positioned near the San Joaquin River, southwest of Madera in Madera County, while the remaining two are in Kern County, southwest of Bakersfield. Detailed descriptions of the location, size, crops, soils, and climate are found in Table 1. The almond trees were 7 to 12 years old and irrigated using drip or micro-sprinkler irrigation. The Central Valley of California has a Mediterranean climate, characterized by hot, dry summers and cool, wet winters. Trees are planted on linear berms that extend 10 to 20 cm above the lanes. The lanes have a cover crop in the winter/spring but are typically cut back in mid-summer to facilitate ground preparation for harvest in late summer. The berms are kept free of cover crops on these sites.

The block KG 18-19 (size: 35 ha) is north of the San Joaquin River, while SSR 35-1 (size: 25 ha) and ST-15 (size: 31 ha) are located adjacent to canals. ST-15 previously had a drainage or canal running through it and was previously part of the adjacent cattle ranch. ST-15 is split into two parts by a gravel ranch road.

## 2. Materials and Methods

### 2.1. Digital Soil Core System and Probe

**Figure 3:**
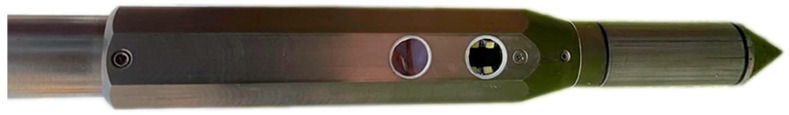
Photo of the Digital Soil Core Probe.

Our research employed the DSC System, which includes the integrated components of the DSC Probe (Figure 3), software, and equipment to deploy. The DSC Probe is a multi-sensor probe that includes (1) tip stress, (2) sleeve friction, (3) dielectric permittivity, (4) electrical conductivity, (5) a microelectromechanical system (MEMS) microphone, (6) a video microscope, and (7) visible and near-infrared (VNIR) diffuse reflectance spectrometers [73]. The DSC Probe can penetrate the soil up to 120 cm in this configuration. Tip and sleeve stress measurements are indicators of the soil strength [74], which is spatially and temporally variable. The DSC Probe incorporates a 60-degree, 1-inch diameter conical tip [5]. A pair of steel electrodes in the tip of the DSC Probe were separated by an insulating element and used to measure the volumetric water content and electrical conductivity via rapidly multiplexed measurements of the direct current (DC) electrical resistance and apparent dielectric permittivity at a frequency above 50 MHz, from which the water content was inferred. The dielectric permittivity of the soil was recovered via calibration to known standards and converted to the volumetric water content (VWC) using well-established relationships [75,76,77]. An embedded microelectromechanical system (MEMS) digital microphone recorded the acoustic emissions produced by the penetration process, as soil particles were scraped and rearranged due to penetration displacement [78,79]. The sound was affected by the soil texture and structure, compaction state, and water content, making the microphone sensitive to several important soil parameters. Two sapphire windows permitted video microscope imagery and VNIR DRS, with optics and lighting optimized for subsurface microscopy at a penetration speed. Uniform, consistent illumination was synchronized to the video frame rate. The videos were captured using the Advanced Video Coding (AVC), H.264, video compression standard. The video was captured in H.264, Red–Green–Blue (RGB) frames and extracted for processing. The microscope produces RGB color imagery (2.3 × 1.2 mm) with a 1-μm pixel resolution and a spatial density of about 15 images per cm, with a 50% overlap of adjacent images. The optical resolution of 3 μm was confirmed using a MIL-STD-150A resolution calculator (#38-257, Edmund Optics, Barrington, NJ, USA). VNIR DRS data were acquired at a rate of four scans per second, with a push rate of 2 cm/s, resulting in approximately 2 VNIR readings per cm. The downhole optical design and proprietary optical fiber bundle of the VNIR system was optimized for a maximum signal-to-noise ratio (SNR) in the spectra collected by the spectrometers located above ground and external to the DSC Probe from Ocean Optics (Orlando, FL, USA, QEPro and NIRQuest) in a custom enclosure engineered for environmental protection and precise thermal control. The QEPro has a spectral range of 350 to 950 nm and full-width half-max (FWHM) optical resolution of 1.2–6.87 nm. The NIRQuest has a spectral range of 900 to 2500 nm and a full-width half-max (FWHM) optical resolution of 6.3 nm. The DSC System includes a string potentiometer used to register the DSC Probe depth during penetration.

In contrast to conventional core retrieval and laboratory analysis, the DSC Probe data collection method preserves the vertical spatial variability, differentiates thin layering, and accurately references the soil parameters to the depth. Other advantages that in situ proximal sensing can provide over the traditional ex situ soil coring, compositing, and homogenization of soil samples include, for example, observing the in situ distribution of soil water within the structural arrangement revealed and the in situ bulk electrical conductivity rather than that of saturated paste extract.

**Figure 4:**
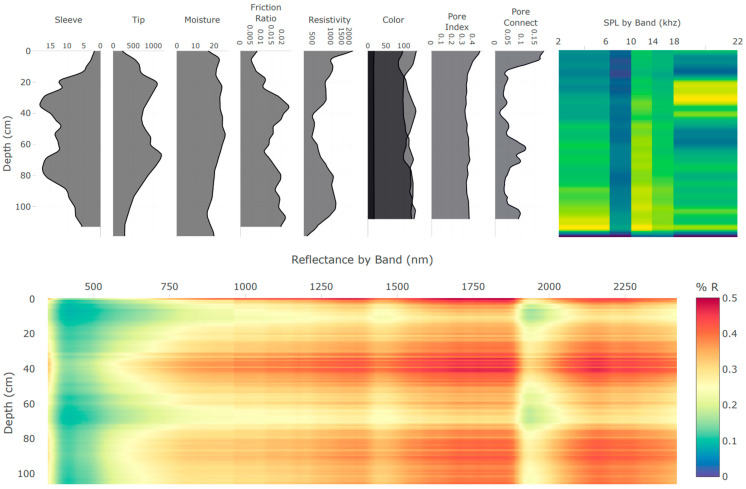
Example of the real-time data acquisition DSC software plots of data features derived from the multiple sensors of the DSC Probe in a single profile collected in about 60 s. Plots are oriented so that the features are aligned by depth on the *y*-axis. Calibrated feature units are scaled to fit the user interface and are not displayed in this example.

**Figure 5:**
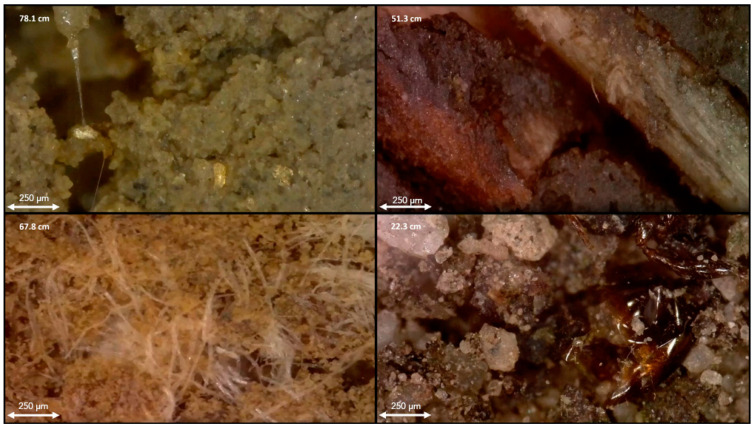
In situ imagery obtained using the DSC, showing (from top left clockwise) microbial gums, roots, mycorrhizae, meso-fauna. Depth from the ground surface is listed in the upper-left corner of each image. A scale bar is in the lower left corner of each image.

The integration of multiple independent proximal soil sensors in the DSC Probe enhances the capacity to capture a comprehensive picture of the soil properties and the in situ relationships to each other. Each sensor modality offers a unique perspective on the soil properties, and, when combined, they provide a multifaceted characterization of the soil profile (example sensor vertical plots are provided in Figure 4 and imagery is provided in Figure 5). An important consideration in the development of the DSC System is the interplay of the orthogonality of the sensor modalities, and the degrees of freedom in the sensor data and soil parameters of interest. Accounting for the dimensionality of information within individual sensors, such as video, audio, and spectrometry, the DSC System provides over 1200 sensor output values for each cm of soil it encounters.

#### 2.1.1. Soil Data Collection

**Figure 6:**
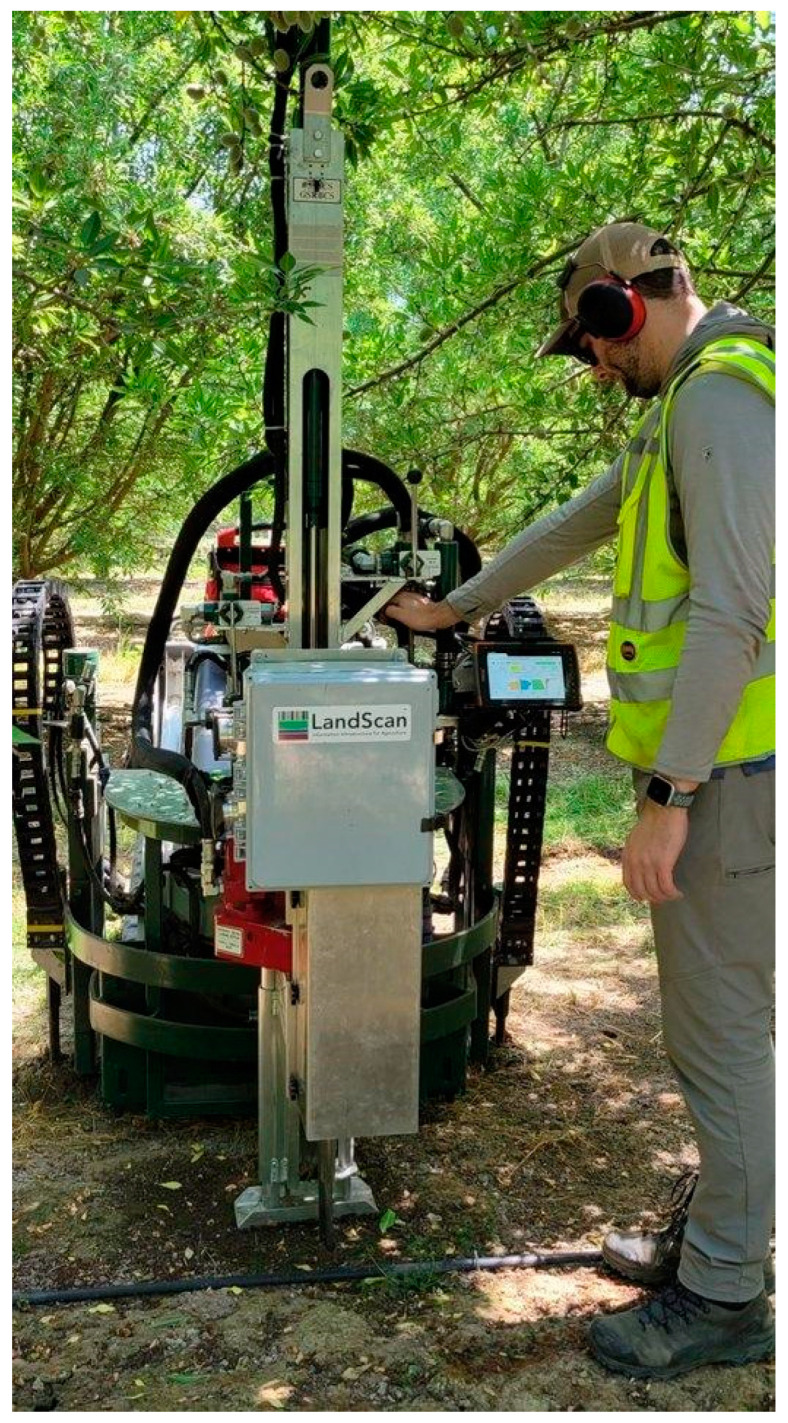
Digital Soil Core System, including the DSC probe, software, and deployment equipment.

The in situ and ex situ soil data collection took place between October and December 2023. Figure 6 shows the DSC System in operation in an almond orchard.

DSC sampling locations and collocated soil cores were targeted using c-means clustering [79] applied to the EM data. The c-means clustering algorithm was used to find six clusters and to identify one DSC target location per cluster. Additional DSC observations were obtained for commercial mapping purposes but were not included in this study.

**Figure 7:**
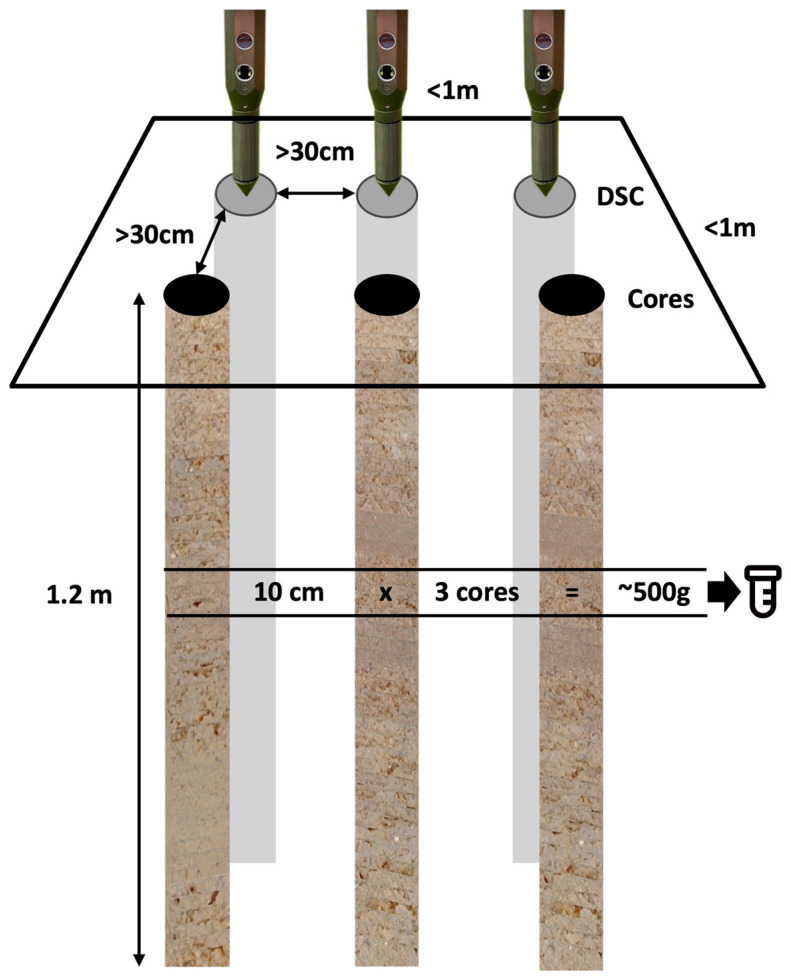
Diagram of the sampling layout. Three in situ DSC System digital profiles and three ex situ soil cores were taken within a 1 m-by-1 m area, designating a sampling site. Cores and DSC digital profiles were at least 30 cm from each other. Samples were taken at 10-cm depth intervals at multiple corresponding depths from 3 cores and combined to make 1 composite laboratory sample that was the equivalent of at least 500 g (or the volumetric equivalent, which equates to at least 10-cm × 3 cores).

Both DSC sensor measurement profiles and physical soil cores were obtained in triplicate at each target location. All were acquired from within an area measuring approximately 1-m-by-1-m at each target location (Figure 7), between the center and the shoulder of the berm on the tree-row berm between two almond trees.

Each DSC Probe measurement profile extended to about 1.2 m below ground, except for the video and spectrometer data, as there was a ~20 cm offset from the tip of the probe to the video and spectrometer window. So, all of the sensors’ data had measurements up to 1 m in depth, which is a widely used depth of investigation in agricultural studies using a penetrometer system [16,80]. Although the topsoil layer (0–30 cm) is the most common and widely used for soil investigations in agriculture, a 1 m depth is considered the root zone depth, which is very useful for long-term soil health assessments and for understanding water infiltration and subsoil conditions, especially in semi-arid and arid regions [81,82,83]. To assure the highest accuracy of the spectral reflectance data, free of instrument thermal drift and other factors, the DSC System automatically performed a series of reference dark current scans at the terminus of every digital profile. With the sapphire window embedded more than 1 m deep in the ground, free from any possibility of ambient light, the illumination source was shuttered, and a dark current reference measurement was obtained. The conversion of the raw spectral scan data to the reflectance spectra considered the nearest-in-time dark current reference scan along with the nearest-in-time white reference scan obtained by covering the sapphire window with a Spectralon^®^ diffuse reflectance standard (Edmund Optics Stock #54-302, Barrington, NJ, USA) and triggering the control software to acquire a series of reference reflectance scans. The processing of the DSC sensor data is described later.

Electromagnetic induction (EM) data were collected along the rows in the almond orchards to help understand the soil variability patterns with a Dualem-1HS (2 distances × 2 orientations), giving 4 channels of apparent electromagnetic conductivity to 4 depths of exploration (30, 50, 80, and 160 cm). The EM was driven down in each row of the mapped orchards. A Real-Time Kinematic (RTK) GPS was used for the georeferencing of the EM data. The data were then processed on-the-fly by using LandScan data collection software to remove the physical and temporal offsets between the GPS and EM, and the vertical offset between the GPS and the ground. The results were filtered using a windowed standard deviation filter and interpolated to rasters using a thin-plate spline algorithm (the Minimum Curvature interpolation algorithm in the Datum Workstation, a geospatial analysis system formally known as TNTmips, LandScan, 2023).

Physical cores were obtained using a 122-cm (48-in) core barrel, with plastic liners having an inner diameter of 41 mm (1.6 in). The soil cores from each location were aligned in a tray, starting at the top and extracted from the plastic tube. Any obvious horizon breaks in the soil were aligned between the cores. The cores were then broken into 10-cm (6-in) horizons across all three cores. Soil that appeared consistent for each horizon (with a volumetric equivalent of at least ~500 g) was bagged for the lab analysis, with 1 bag per horizon across 3 cores. Three cores were used to keep the horizon thickness small while providing a sufficient sample volume to the lab. Samples beyond 110 cm (42 in) were not sent to the lab. Soil samples were labeled with DSC push identification numbers (IDs) so that they could be matched to the DSC sensor data for training.

In total, 60 soil cores and 60 DSC digital profiles were collected within the whole study area. Refer to Table 1 for the number of soil samples submitted by study area. Approximately 6–8 sampling depths were selected from each ex situ soil core location and sent to a commercial laboratory (Dellavalle Laboratory Inc., Fresno, CA, USA) for analysis. Soil analytical measurements included the organic matter (OM, loss on ignition), particle size (sand, silt, and clay measured by the hydrometer method), and a complete soil fertility package. Out of the measurements in the soil fertility package, boron (B), calcium (Ca), copper (Cu), zinc (Zn), and the pH were evaluated in this study. References to the laboratory methods used are included in Table 2. Note that nitrogen (N) was excluded because the concentrations at each site were very low, with no data distribution to measure against.

#### 2.1.2. Crop Data Collection

For this study, the almond crop vegetation was characterized utilizing the Digital Vegetation Signature ^TM^ (DVS) technology developed by LandScan [84]. Each site was flown mid-season for the study with a DGI Mavic M3M multispectral unmanned arial vehicle (UAV) at an altitude of 120 m. The UAV has an RGB camera, a multispectral camera, and a built-in GPS. The imagery was processed using the Rig Camera Alignment tool in the Datum Workstation. A spectral calibration was performed against ground control targets prior to mosaicking in the Datum Workstation, which was then used to produce the final orthorectified mosaics. The mosaics were processed into a vegetation vigor index (VVI), a pigmentation index (PI), and numerous other indices using proprietary algorithms in the Datum Workstation. The richness of the combination of both spectral and spatial data reveals many new features in and about the data that provide valuable input to future analytical processes and integration into the LandScan Digital Twin for Agriculture [85].

**Figure 8:**
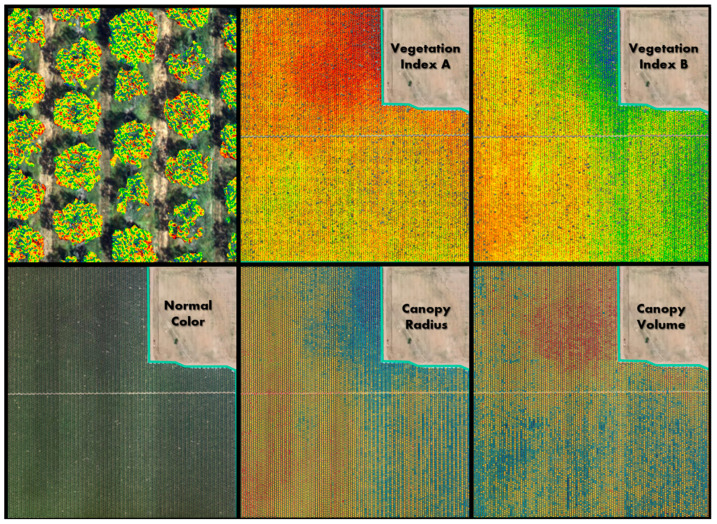
DVS data from the same flight shows how different crop metrics produce different patterns indicating unique spatial information. The top left panel shows a zoomed in view of Vegetation Index A, clipped to the tree canopy and overlain on the natural color image. The colors of Vegetation Indices A and B, Canopy Radius, and Canopy Volume show relative values using a spectral color ramp with red as the lowest values and blue as the highest values.

In addition to the orthorectified imagery, the orthorectification process also resulted in a digital surface model (DSM). The digital surface model was used in conjunction with a digital terrain model (DTM), acquired from the U.S. Geological Survey (USGS) National Elevation Dataset program, along with a vegetation raster to create a vegetation height raster. The vegetation height raster was used to determine the location of each tree in each orchard block, give them an identifier, and establish various canopy masks. One example is that, for each pixel in the canopy mask, the height was multiplied by the VVI and then summed to form a total Crop Productivity Index (CPI). This approximates the canopy volume and density, or the total canopy biomass (e.g., volume × density should equal mass), which relates to the fractional amount of photosynthetically active radiation (fPAR) that can be absorbed by each tree. In theory, an almond tree’s productive capacity is limited by the fPAR [86]. The DVS data collection process resulted in a finite number of ‘named’ indices, but also produced many new data features and relationships that empowered deeper learning opportunities for advanced analytics (Figure 8). Many of these features and relationships were integrated into the digital twin for exploratory and discovery purposes as additional agronomic metrics became available. These data enable a wide range of opportunities to advance and improve on the approach taken in Method D in this study.

### 2.2. Data Pre-Processing and Harmonization

The multi-sensor data that were collected with the DSC System were screened to identify outliers, noise, and missing data as part of the quality assurance procedures in LandScan’s DSC data collection software. Data collected from all three profiles were then merged spatially to obtain one representative digital profile to integrate with the soil properties and crop responses for modeling purposes.

### 2.3. Spectral Data Processing

Using reference standards and dark current measurements, as described above, spectral reflectance from the DSC Probe was first computed. In this work, reflectance spectra were converted to absorbance spectra before applying Standard Normal Variate (SNV) Transformation and Savitzky–Golay (SG) filtering [87]. The SNV minimizes multiplicative effects such as baseline shifts and light scattering in spectroscopic data [88]. SG filtering was applied to remove noise and improve the signal-to-noise ratio of the spectral data while preserving the spectral features. For this purpose, 1st differential order and 2nd polynomial order with 11 window sizes were used. Spectral pre-processing was performed using the prospectr package in R, version 0.2.7 (https://CRAN.R-project.org/package=prospectr, (accessed on 1 January 2024).

### 2.4. Processing of Digital Soil Images

Image color metrics, the mean hue, value, and saturation (HSV), were extracted from each DSC Probe microscope image, as well the succolarity, a metric of the image structure (de Melo et al., 2008), for consideration in the analysis. Of these image metrics, the succolarity curve difference, color saturation, color hue and color value were found to have significance in the final ML model. Succolarity was originally developed to measure the flow of water through canal systems from satellite images [89], with additional flow-related applications suggested by de Melo and Conci [90]. LandScan applies succolarity algorithms to quantify the potential for percolation flow through porous media in an image in the analytics software. The determination of the succolarity begins with the binary masking of the image based on a threshold value below which a pixel is considered to represent a void (the pore space) and above which a pixel is considered to represent a structure (the soil matrix). The binary image is then flooded with a theoretical ‘fluid’ from each of the four edges of the image boundary, and the proportion of the total image penetrated by the fluid from each direction of flooding is computed. The four values are then averaged into a single succolarity value. This approach to computing the succolarity, by Leavitt et al. (2021) [91], approximates the methods explained in de Melo and de Melo and Conci [89,90]. Since the succolarity value thus computed is a function of the threshold chosen for the binary masking operation, we generated multiple values of the succolarity as a function of the threshold value chosen, which comprise a succolarity curve. This curve tends to exhibit a sigmoidal shape, and the metric we call the succolarity curve difference is the normalized difference in the image masking thresholds between the start and end of the rise in the succolarity curve [91].

### 2.5. Processing of Audio Data

Audio data from the MEMS microphone was recorded in Waveform Audio Format (WAV) for storage. The WAV file was processed in Python by converting it to a numpy array and running a 3-kHz high-pass Fourier transform filter, followed by binning into five bins of a 4-kHz bandwidth, a sound pressure level for the band, and the total sound pressure level.

### 2.6. Processing of Other Sensor Data

All DSC System sensor-derived data were harmonized to co-registered 1-cm depth increments in the LandScan DSC processing software. The outputs of all DSC Probe sensors and the DSC System string potentiometer were used to register the DSC Probe depth during penetration, and are each associated with a time stamp during the data acquisition. Because the sensors and their contact with the soil each occupy a different position along the DSC Probe as it advances through the vertical profile, each increment of soil is encountered by a different sensor at a slightly different time. To co-register the readings from all of the sensors with respect to the depth, given that slight variations in the penetration speed may occur during the acquisition of a sensor profile, each time series of sensor readings was first independently indexed to the depth and then re-sampled relative to a common index of equally spaced depth intervals, such as every 1-cm. Depth co-registration was achieved by applying a sensor-specific depth offset to each sensor in the probe based on its relative position in the DSC Probe, then computing the depth each sensor was at when each of its readings were recorded, then re-sampling the readings from each sensor independently using cubic spline interpolation to conform to a uniformly spaced set of depth values distributed over the depth of the profile with a depth referenced to zero depth at the ground surface.

### 2.7. Data Feature Selection

The Boruta feature selection algorithm was applied to reduce the dimensionality of the massive data cube of the sensor data by identifying the most relevant sensor output for predicting the soil properties (Methods A, B, and C) and crop responses (Method D). It is one of the widely used variable selection methods in soil spectroscopy to deal with the multi-collinearity of data [92,93,94]. Boruta trains an RF model using a combined dataset of original and shuffled features, and evaluates the variable importance (Z score) for each predictor. Then, it checks whether a real predictor has a higher importance (RMSE) than the best of its shadow predictors to decide on the important and unimportant features. In this study, all of the high-resolution (1 cm) sensor data were used as features data in the RF classifier from the Scikit-Learn library in Python to select the important features for individual soil properties and crop responses [95].

### 2.8. Comparison of Training Methods

**Figure 9:**
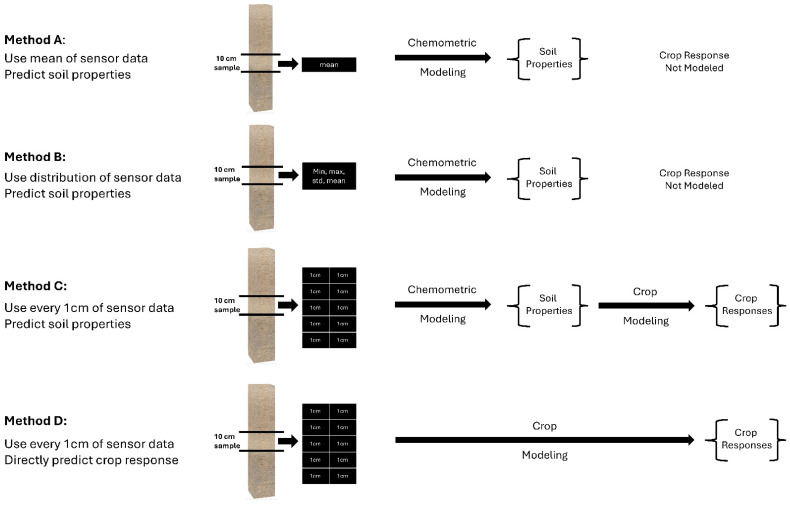
Illustration of the four methods to pair-up the in situ DSC System data and ex situ soil analytical data (Methods A, B, and C), and the DSC System data and DVS crop data (Method D). Method C was chosen to compare to Method D for modeling the direct crop responses.

Four different methods were used to assess the model performance of the soil health and management properties. Modeling was performed with the PLSR using leave-one-out cross-validation [96]. The goal was to determine the best method of assessment between Methods A, B, and C as compared to the laboratory, and then use that method to compare to Method D in predicting the crop response (Figure 9).

Method A reduced the high-resolution DSC Probe sensor data to discrete values paired with layer-based soil laboratory measurements. All of the high-resolution (1-cm) sensor data were averaged to match the length of the segments of the ex situ soil cores sent to the laboratory for analysis. In essence, for each laboratory measurement, one array of DSC Probe sensor data was used in the calibration models. We used the leave-one-out cross-validation method and PLSR on the sensor and soil analytical data from each of the 15-cm layer increments for all cores.

Method B used stochastic distributions of the DSC Probe sensor data paired with layer-based ex situ soil laboratory measurements. Here, the stochastic distributions of all DSC Probe sensor data in the model were used for the PLSR modeling. In this method, soil analytical laboratory data were matched with the minimum, maximum, standard deviation, and mean sensor data associated within a 15-cm layer. For the validation, the arrays of the minimum, maximum, standard deviation, and mean DSC Probe sensor data for the corresponding soil analytical laboratory samples were used in the leave-one-out cross-validation.

Method C allocated the same soil analytical laboratory data to each one of the high-resolution multi-sensor data within a layer. PLSR models used all of the high-resolution (1-cm) DSC System data corresponding to the soil analytical laboratory measurements. Since the laboratory measurements were only available for each layer, the same laboratory data values were matched with all of the corresponding high-resolution DSC System data. For the validation, however, we ensured that, for each laboratory sample left out during the cross-validation, every high-resolution DSC System data increment corresponding to the sample that was left out was also left out. The predicted soil properties were averaged for each 15-cm layer.

Method D linked the high-density in situ DSC System data directly to the DVS crop responses (the crop performance and behavior metrics), bypassing costly laboratory soil analysis. In this approach, the DVS crop responses, such as the Crop Productivity Index (CPI), canopy area, and canopy volume, were directly predicted from the DSC System to avoid the laboratory measurements of the soil properties. Since crop responses are single measurements of each location, the optimum depth for aggregating the sensor data was determined. A few different soil depth intervals (0–20, 0–30, and 0–60-cm) were considered to find out the optimum depth of the DSC System data that predicted the crop response with comparatively higher accuracies. Finally, based on the soil health and nutrient management opportunities in the almond trees, the 0–30 cm depth was considered for the analysis. The same crop response was matched with the array of high-resolution (1-cm) DSC System data for training and validating purposes. Then, the predicted crop responses were averaged and compared with the observed DVS CPI, canopy areas, and canopy volumes.

### 2.9. Modeling Approach

PLSR modeling has been a workhorse in digital soil mapping and one of the most robust machine learning methods [97,98]. The PLSR with the leave-one-out cross-validation approach was used to estimate the soil properties (Methods A, B, and C) and crop responses (Method D). The important features of each soil property were used to train the individual models to estimate that property. All of the samples from the individual fields were used for training the calibration model, except one sample that was used for validating the calibration model. The number of components (n-component) used to obtain the lowest RMSE between the measured and estimated soil properties and crop responses in the training model was used for validation purposes. The n-components provide the fitting between inputs and outputs. The more n-components are used, the more complex are the relations between the input and output variables that can be modeled. Modeling was performed using the Python programming language with “PLSRegression” from the scikit-learn 1.2.1 package. For evaluating the performances of all four methods, the R^2^, RMSE, RPIQ, and bias of the modeling were used.

## 3. Results

### 3.1. Feature Selections for Modeling

**Figure 10:**
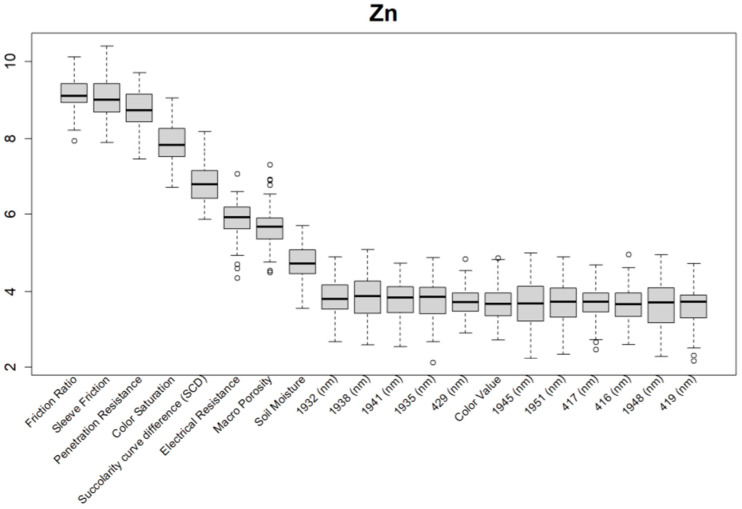
Boruta variable importance graph for the soil properties (Method C), showing the top 20 DSC Probe sensor features for the prediction of zinc (Zn).

**Figure 11:**
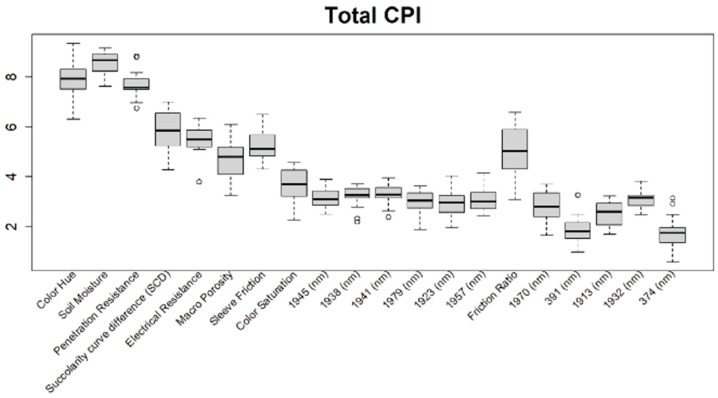
Boruta variable importance graph for the crop performance property CPI (Method D), showing the top 20 DSC Probe sensor features for the prediction of CPI.

Before training the predictive models for the estimation of the soil properties, the Boruta feature selection algorithm was applied to over 1200 features per cm in the DSC System dataset to obtain the importance of the sensor data that will be used in the models. The top 20 important features of soil properties and crop responses are shown in Figure 10 for zinc (Zn) and Figure 11 for the CPI. The other Boruta graphs can be found in Appendix A. For both the soil properties and crop responses, all of the features that had a Z score of more than the maximum shadow value were used for the predictions. The range of features used in the soil property predictions in this study varied from 36 to 83.

For almost all soil properties, the DSC Probe sleeve friction, penetration resistance, friction ratio, and 1600–2000 nm wavelengths from the VNIR spectra were found to be the most important features. From the whole VNIR spectral range, the near-infrared (NIR) wavelengths seemed to be important for all of the soil properties except for Ca.

For the crop responses, the color (hue), color (saturation), color (value), soil moisture, penetration resistance, succolarity curve difference (SCD), electrical resistance, macro-porosity, sleeve friction, and friction ratio were the important features, along with several bands from the VNIR spectra. Similar to feature importance for the soil properties, the NIR region had more important features compared to the visible region of the spectra for the crop response.

### 3.2. Predictive Accuracy of Soil Properties Modeling Methods

**Figure 12:**
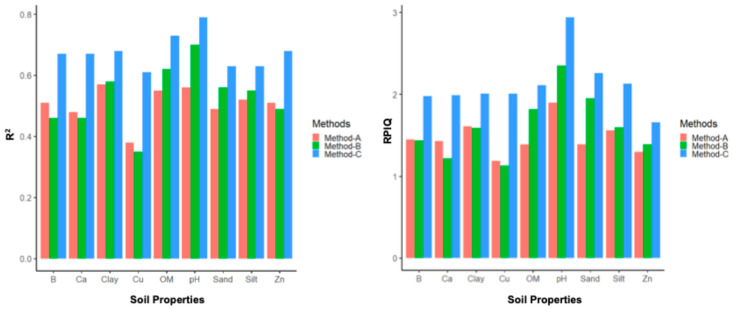
Mean R^2^ (**left**) and mean RPIQ (**right**) for each method for each soil property summarized across the study sites.

This study compared the prediction capabilities of three different methods for modeling various soil properties (OM, sand, clay, silt, B, Ca, Cu, Zn, and the pH) using DSC digital soil profiles. The results for the R^2^ and RPIQ are summarized by property in Figure 12, and more detail on the R^2^, RMSE, bias, and RPIQ per study location and property are reported in Appendix C.

Method C had the highest R^2^, the lowest RMSE, and the highest RPIQ across all nine soil properties at all three sites. It also had the highest mean R^2^, lowest mean RMSE, and highest mean RPIQ across the three sites for all nine soil properties, indicating that Method C leads to more accurate and robust results than the other two methods. When the high-resolution (1-cm) sensor data were averaged to correspond with the length of the soil sample segments analyzed in the laboratory for determining the soil properties, the correlation between the sensor data and lab results decreased due to the averaging process. So, Method C performed much better than Method A. Method B only slightly outperformed Method A for some properties (OM, sand, clay, silt, and the pH), but not all properties (B, Ca, Cu, and Zn), as it included additional stochastic distributions in the DSC sensor data (the minimum, maximum, standard deviation, and mean). But the prediction accuracies of Method C were still better than Method B, as this method was able to train the model with sufficient variation in the DSC sensor data that correlated with the corresponding lab measurements of different soil properties.

### 3.3. In Situ DSC System to Ex Situ Laboratory Properties to DVS Digital Crop Performance vs. DSC System to DVS Digital Crop Performance

We compared the performance of two models that predict crop performance from in situ and ex situ soil data. In the first approach, we used the best-performing method for predicting the soil properties, Method C, and predicted the soil properties, and then used the soil properties to predict the CPI, canopy area, and canopy volume as measured by a UAV across all three sites.

In the second approach, we directly predicted the CPI, canopy area, and canopy volume based on the in situ DSC System data directly, without predicting the ex situ laboratory soil property values (Method D). For the CPI, Method D had an R^2^ between 0.72 and 0.75, and an RPIQ between 1.13 and 1.64 (Table 3), whereas the prediction of the CPI using Method C had an R^2^ between 0.54 and 0.67, and an RPIQ between 0.63 and 0.85. Method D had a higher R^2^ and a lower RMSE than Method C, indicating that the in situ DSC System data (input data) to digital crop response models show a higher accuracy than a more complex approach that sequentially models the in situ DSC System data (input data) → soil properties → crop response models.

## 4. Discussion

The results show that the sensor data collected from the in situ DSC System has the potential for estimating the soil properties and crop responses with the support of chemometrics modeling. All three methods (Methods A, B, and C) used in this study showed some correlation between the DSC System data and various soil properties, but Method C exhibited the highest prediction accuracies compared to the other methods. The RPIQ for Method C ranged between 1.66 (Zn) and 2.94 (pH), which are compelling results for soil predictions using the in situ DSC System data. The R^2^ for the soil properties (Method C) ranged from 0.61 (Cu) up to 0.79 (pH), and 0.73 (OM), suggesting improved results compared to other proximal soil sensor applications. For example, models to predict the SOC stock using field VNIR spectral data in a study in France achieved an R^2^ between 0.52 and 0.86, and an RPIQ between 1.61 and 4.49, in validation mode [51]. On experimental plots in Canadian provinces with a humid soil moisture regime, the SOC concentrations modeled by VNIR spectra achieved an R^2^ of 0.54 (MIR) and an R^2^ of 0.49 (VNIR) [52]. In a study in Germany, the topsoil SOC (%) predictions (validation mode) using a Veris full-range VNIR device and PLSR modeling showed a modest R^2^ (0.55) and RPIQ (2.05), the Hamamatsu sensor showed poor performance, with an R^2^ of 0.29 and an RPIQ of 1.67, while the NeoSpectra results were slightly better, with an R^2^ of 0.48 and an RPIQ of 2.00 [27]. Many soil sensor applications focus only on the predictions of the SOC or OM, while the DSC System presented in this study has a much broader range to model a suite of different soil properties.

One of the major advantages of the DSC System is the high resolution (<1 cm) of data acquisition from all of the sensors. In Method A, the mean of all 1-cm data in a horizon was used to train and validate the model. For Method B, instead of only using the mean data, the minimum, maximum, standard deviation, and mean of all sensor data in a soil layer were used. However, in Method C, the full potential of the high-resolution DSC System data was used, which allowed for the training of the model in retaining the variation in the sensor data along the soil profiles. The higher prediction accuracies from Method C for all soil properties indicate the importance of recording high-resolution sensor data for accurate soil predictions.

From the Boruta feature selection (Figure 10 and Figure 11; Appendix A), it was observed that most of the important features were obtained from the VNIR spectral ranges, specifically for the soil textures (clay, silt, and sand), OM, and pH. The prediction accuracies for these properties were better compared to the other soil properties estimated using the DSC System. Other DSC Probe data, such as the sleeve friction, penetration force, friction ratio, and color saturation, were the most important features, as these appeared in most of the Boruta important feature plots. Several features, such as the succolarity curve difference, electrical resistance, macro-porosity, and soil moisture, were also found to be important for the estimation of the pH and Zn. Most of the important features were found from the VNIR spectral ranges for the nutrients, except Zn. For the crop responses, all of the color properties (hue, saturation, and value), the penetration resistance, the sleeve friction, the friction ratio, the soil moisture, and the micro-porosity were found to be the most important features, followed by the VNIR spectra.

It is practically not possible to obtain an objective, spatially accurate map if ex situ soil sampling is utilized on large farming operations (large fields and ranches). Conventional wet chemistry analyses involve extracting soil cores from the field, transporting samples, and processing samples for laboratory analyses. Maintaining all of these standard protocols disturbs the original condition of the soil samples [99,100]. This study brings into serious question the accuracy and applicability of conventional ex situ soil sampling and laboratory practices for advanced agronomic analytics, and negates the opportunity to produce a digital twin. Many human (e.g., the handling of soil samples or cores) and laboratory measurement errors may occur without even acknowledging and quantifying them explicitly.

This study shows that multi-sensor data collected using the DSC System can rapidly and objectively estimate multiple soil properties. All of these data were collected in situ and within a fraction of the time for extracting ex situ soil cores in the fields, and tested in the laboratory using wet chemistry analyses. Importantly, the DSC System reduced the time and cost of characterizing the soil profile by reducing or even omitting the expenses for extracting the ex situ soil cores, processing them, transporting them to commercial soil testing laboratories, and performing wet chemistry analyses.

Since the density of the DSC System data (<1 cm scale) is substantially higher than discrete sample extraction in different soil layers by traditional soil analysis in the laboratory, one may argue that the real “gold” standard are the DSC System data. These sensor data are collected in close proximity to the soil matrix under actual field conditions, providing a more direct way to characterize soils than conventional ex situ soil surveys. Therefore, our study lays the foundation to shift the paradigm of future soil sensor applications to focus directly on sensor data (e.g., VNIR hyperspectra and porosity derived from digital micro-images) and crop responses (e.g., canopy density) rather than soil interpretations (e.g., OM or soil texture).

In this study, DSC System data were successfully used to estimate crop responses in all three fields (Method D). Bypassing the estimation of the soil properties to estimate the crop responses directly from the sensor data can potentially offer a more streamlined, objective, efficient, and accurate approach to precision agriculture. This approach avoids the risk of errors associated with the indirect, subjective, and analog measurements of some soil properties that are not related to the sensor data used for modeling. Directly estimating the crop responses from the soil sensor data simplifies the data processing pipeline by eliminating intermediate steps (e.g., soil property predictions that match laboratory measurement methods), leading to faster and more efficient data analysis that can be used for the precision management of crops.

## 5. Conclusions

We demonstrated that the DSC System makes robust soil property predictions across multiple soil properties and study areas in central California using a standard machine learning approach. The use of the technology in other crops and soil regions, and the applications of advanced ML algorithms, will further improve sensor-driven soil and crop modeling approaches that promise substantial future cost savings. The innovative DSC System facilitates the collection of standardized soil signatures from multiple concurrent sensing modalities that are spatially co-registered within specific soil profiles.

The integration of the DSC System’s multiple sensing modalities better conditions the ML model solutions that infer specific soil properties or soil–crop relationships from the sensor data. This provides a predictive performance that is superior to sensor systems with fewer modes, such as proximal soil sensors that use diffuse reflectance spectroscopy alone, or with fewer complements. Each additional sensor modality added confidence. In addition, integrated multi-sensor data collection from a single device is less error-prone than multiple single-sensor systems used in combination, which suffer from sensor displacement and disharmonious sensor resolutions that require more extensive post-processing of data to correct and more extensive soil sampling to support.

The best method of training a model to predict soil laboratory data was Method C, which involves treating each centimeter of sensor data as a separate measurement that is paired with a soil lab measurement. Our results suggest that the machine learning algorithms can learn more from a high density of sensor data retaining the spatial variation in soil characteristics along a soil profile compared to aggregating sensor data to a coarser scale (i.e., collecting a soil sample representing a soil layer that is then analyzed in the lab) to match conventional soil surveys. The robust model performance underpins the importance of the vertical scale when characterizing soil properties with multiple sensors, outperforming the traditional soil surveys. The collection of in situ sensor data in soils is a prerequisite to create realistic digital soil twins, which cannot be achieved with soil core extraction and/or through conventional soil laboratory analysis. Thus, we envision a new technology-informed “gold” standard for digital soil mapping, employing a multi-sensor in situ proximal sensor suite combined with AI modeling rather than the traditional standard of discrete soil sample extraction and ex situ analysis.

The best method of training a model to predict crop productivity was Method D (sensor data → crop responses), which outperformed the more complex approach using sequential modeling (sensor data → soil properties → crop responses). These results suggest that direct sensor–crop modeling has fewer errors and higher accuracies than sensor–soil-crop modeling, which suffers from error propagation, lowering the overall model performance. From a statistical perspective, clearly the path of sensor data → crop responses modeling is preferable. The analytical potential of combining a full DSC digital soil profile as a source of calibration for drones, airplanes, and satellite data is compelling.

While the direct prediction of crop productivity could be useful in determining the productivity potential, and inform and improve certain agronomic practices, soil property prediction will still be valuable for describing the below-ground factors that affect that potential. In turn, this helps to determine what actions a grower can take to improve crop productivity in their fields, such as the production of variable rate (VR) fertility and soil amendment maps, or adapting irrigation practices to optimize based on soil variability.

Multi-modal in situ proximal soil sensing systems such as the DSC System present immense potential to transform soil–crop digital mapping and modeling. We continue to acquire DSC System data and corresponding ex situ soil cores for laboratory analysis from numerous locations in California and other locations (e.g., Australia). All of the samples with ground-truth data are being used to develop a DSC data library, which is currently being used to train ML models using data that comprise large variations in soil properties and conditions (e.g., moisture content, fertility, etc.). Over time, we plan to expand the DSC data library with ex situ soil samples and measurements across the U.S. and major agricultural areas world-wide. The goal is to reduce and eventually eliminate the need to extract soil cores from every field. A sufficient quantity of data will enable the application of more data-hungry, deep learning models that will use the diverse and extensive dataset for training the prediction models more efficiently as new soils are added to the library. The DSC data library will enable the exploitation of the full potential of the DSC System’s speed, cost, and reproducibility advantages by estimating soil properties and crop responses from any field in the future using only DSC data. These developments are essential to inform decision support systems that truly optimize climate-smart agricultural management, high-accuracy soil carbon accounting, precision agriculture applications, and the installation of management-unit-level digital twins. Looking forward, this high-spatial-and-information-density data cube will be the type of input necessary to run quantum computing models for future agricultural decision support, particularly in intensively managed cropping systems facing resource constraints.

## Figures and Tables

**Table 1:** Study blocks. Soil units sourced from the United States of Department of Agriculture (USDA) Web Soil Survey, accessed in June 2024.

Block	Location	Description	Samples
KG-18-19	About 20 km southwest of Madera and less than 1 mi north of the San Joaquin River in Madera County, California	A 35.2 ha almond orchard, planted in 2017. Double-line drip irrigation. Soil map units are El Peco-Dinuba fine sandy loams and Grangeville sandy loam, with 0–1 percent slopes (leveled during planting).	78 samples December 2023
SSR-35-1	About 8 km southwest of Bakersfield in Kern County, California	A 25.5 ha almond orchard, planted in 2012. Micro-sprinkler irrigation. Soil map units are primarily Kimberlina fine sandy loam with a small section of Granoso loamy sand adjacent to canal, with 0–2 percent slopes (leveled during planting).	36 samples October 2023
ST-15	About 18 km southwest of Bakersfield in Kern County, California, and about 3 mi south of SSR-35-1	A 31.2 ha almond orchard, planted in 2016. Double-line drip irrigation. Soil map units include Garces loam, Kimberlina fine sandy loam, Millox clay loam, and Tennco fine sandy loam. The field is split into two sections by a field road. The western section is adjacent to a canal.	34 samples October 2023

**Table 2:** Soil analytical measurements performed on the samples in this study. See the NAPT manual for detailed method descriptions (NAPT, 2013).

Property	Abbrev.	NAPT Method	Units	Method Comment
Organic Matter	OM	S9.20	%	Loss on ignition
Sand	Sand	S14.10	%	Hydrometer
Silt	Silt	S14.10	%	Hydrometer
Clay	Clay	S14.10	%	Hydrometer
Boron	B	S1.50	mg/L	Saturated paste
Calcium	Ca	S5.10	mg/kg	AA extraction
Copper	Cu	S6.10	mg/kg	DTPA extraction
Zinc	Zn	S6.10	mg/kg	DTPA extraction
pH	pH	S1.10	pH units	Saturated paste

**Table 3:** Model evaluation metrics for all crop responses (Methods C and D) directly from the DSC System variables as inputs into the PLSR model.

	**Method C**											
	**CPI**				**Canopy Area (m^2^)**				**Canopy Volume (m^3^)**			
**Fields**	**R^2^**	**RMSE**	**Bias**	**RPIQ**	**R^2^**	**RMSE**	**Bias**	**RPIQ**	**R^2^**	**RMSE**	**Bias**	**RPIQ**
St-15	0.67	6.34	−0.07	0.63	0.67	4.79	−0.15	0.58	0.68	14.56	−0.06	0.7
SSR-35-1	0.66	20.97	−0.47	0.75	0.58	3.73	0.02	0.65	0.63	25.35	−0.16	0.81
KG-18-19	0.54	10.32	−0.01	0.85	0.44	2.13	0	0.58	0.48	15.74	−0.06	0.68
	**Method D**											
	**CPI**				**Canopy Area (m^2^)**				**Canopy Volume (m^3^)**			
**Fields**	**R^2^**	**RMSE**	**Bias**	**Fields**	**R^2^**	**RMSE**	**Bias**	**Fields**	**R^2^**	**RMSE**	**Bias**	**Fields**
St-15	0.75	5.09	−0.09	1.13	0.76	3.65	−0.18	1.03	0.76	11.51	−0.19	1.16
SSR-35-1	0.74	17.93	−0.41	1.27	0.72	2.94	0.01	1.06	0.73	21.2	−0.15	1.21
KG-18-19	0.72	8.15	−0.08	1.64	0.65	1.72	−0.01	1.33	0.70	12.23	−0.11	1.55

## Data Availability

The datasets used in this study were acquired from commercial farming operations and may be made available by the authors on request, pending the approval of all parties.

## Appendix A

Additional Boruta variable importance graphs for both soil properties and crop responses, showing the top 20 significant DSC features that were used in the model.

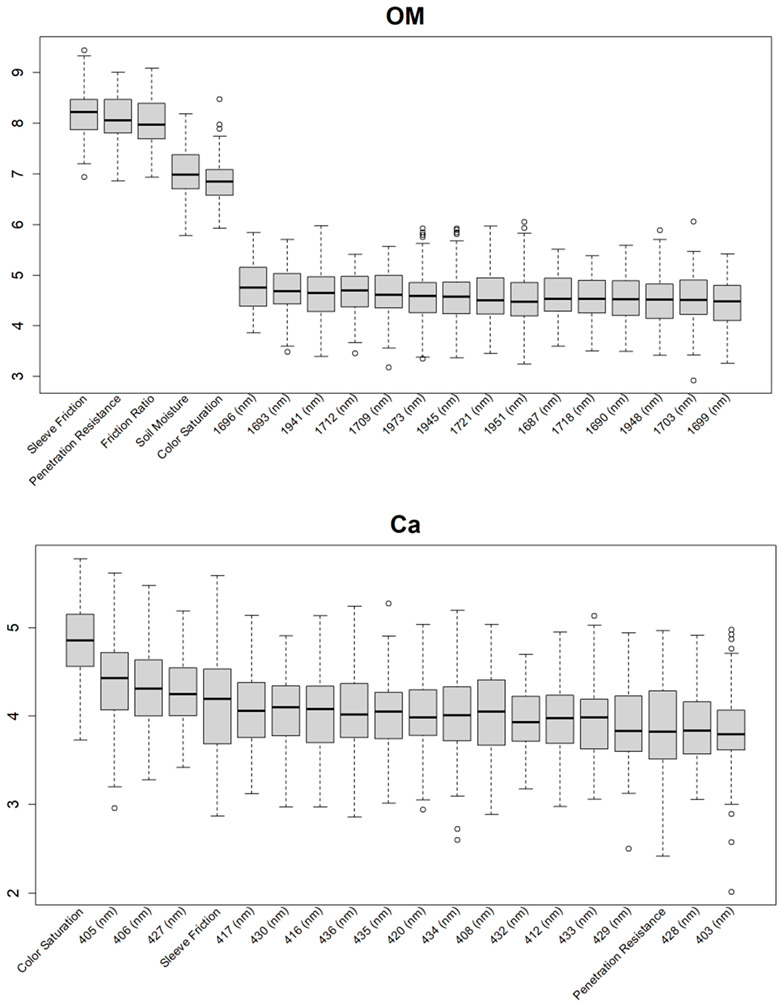
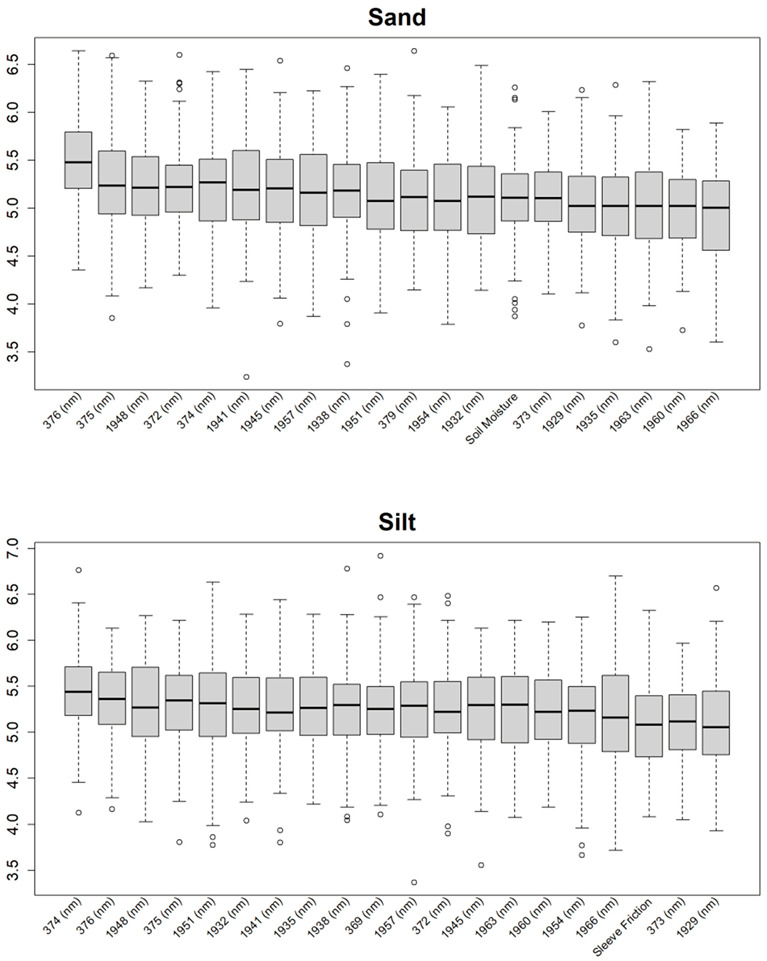
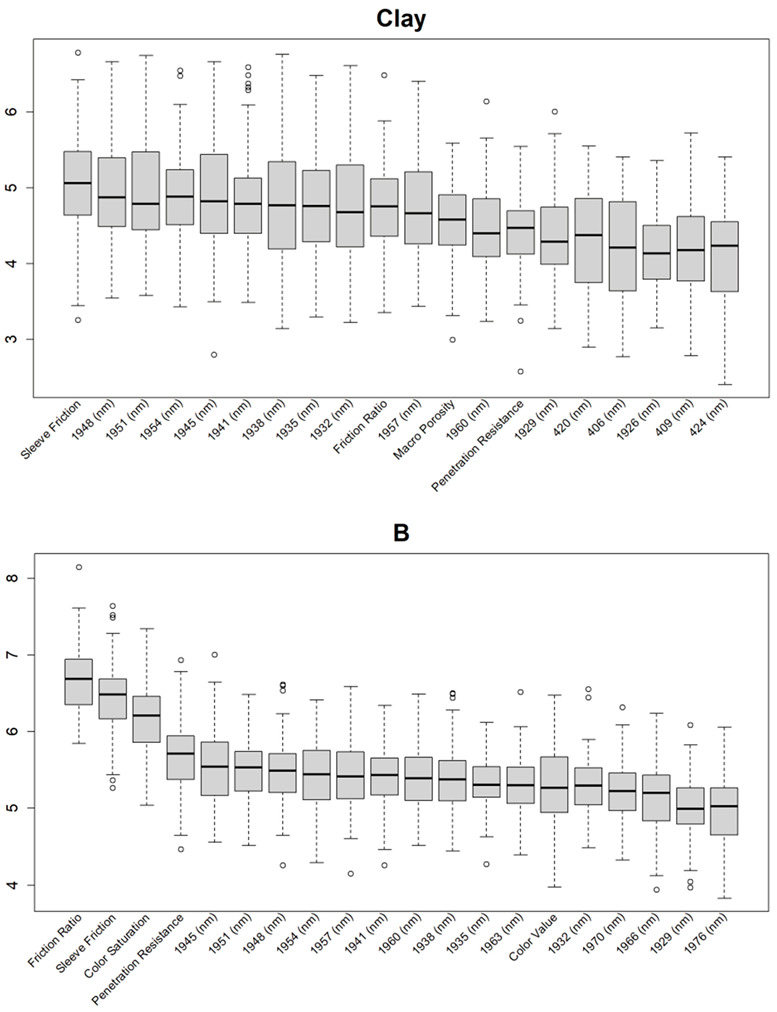
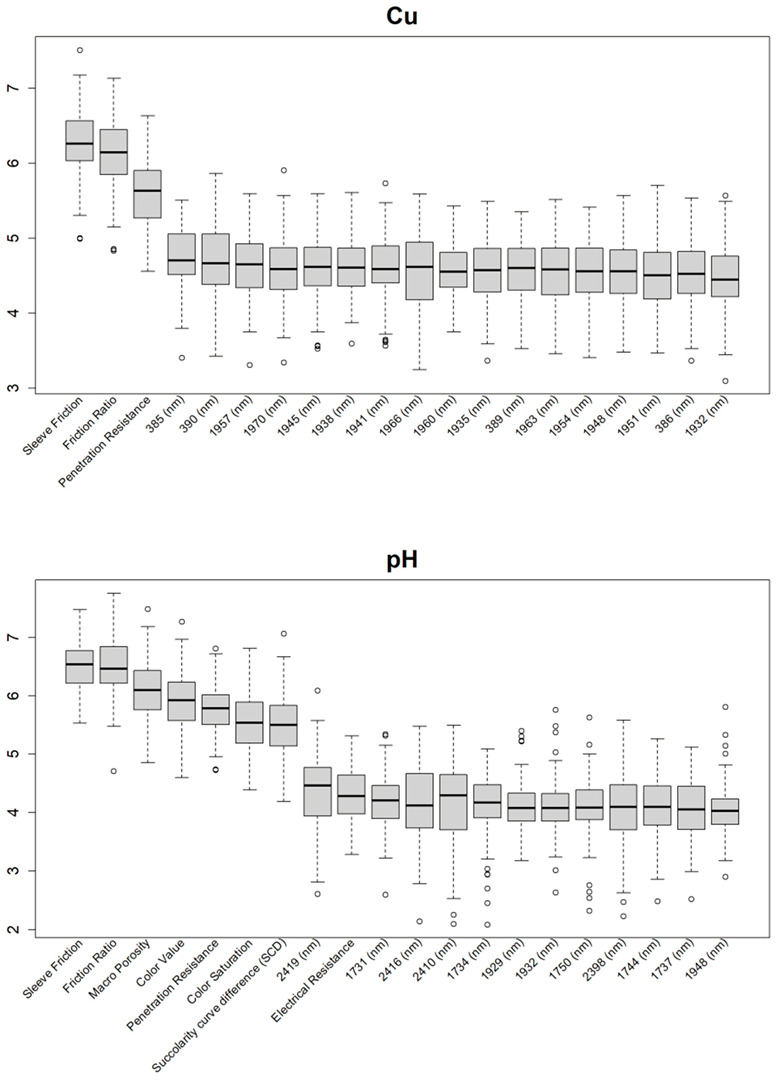
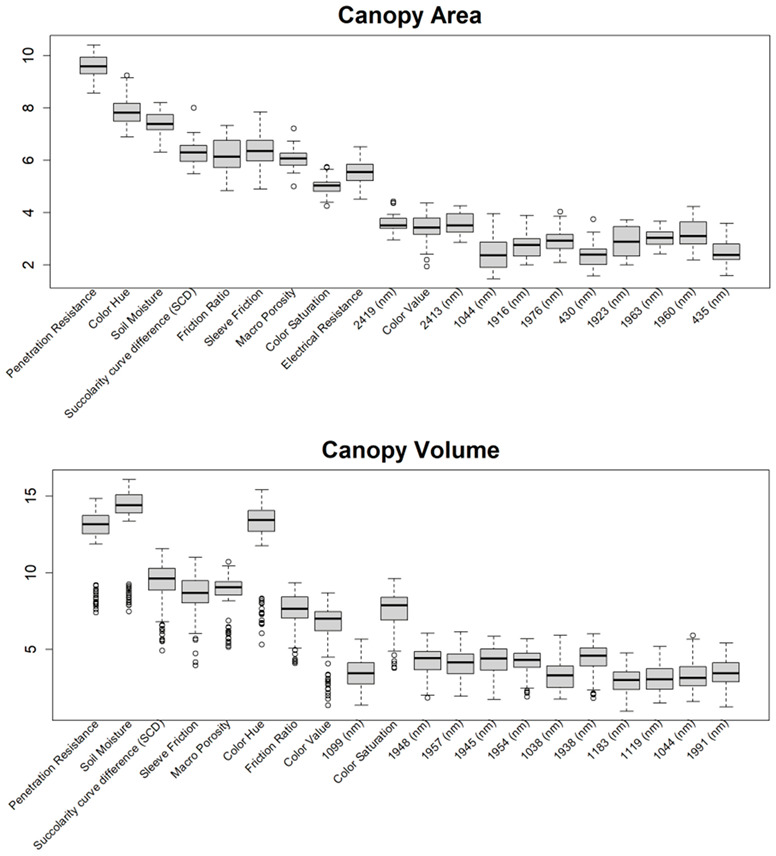

## Appendix B

Histograms of all modeled variables.

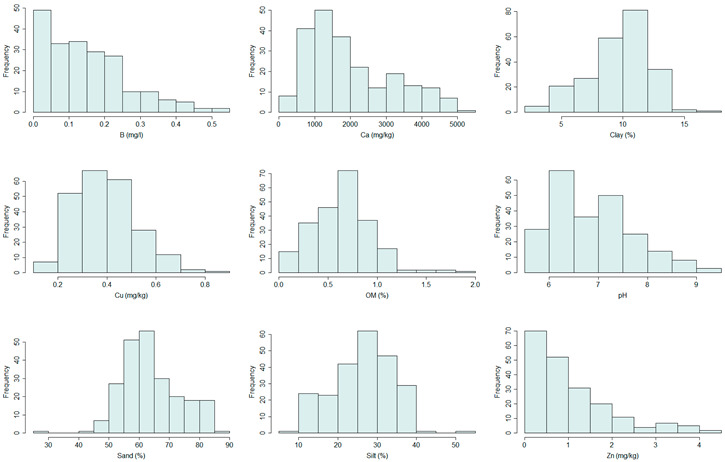

## Appendix C

Model evaluation metrics for the ‘Ex situ Laboratory vs. In situ DSC System’ for all soil properties (Methods A to C), with sensor variables as inputs into the PLSR model.

**Table:**

Methods		A				B				C			
Field	Metrics	SSR 35-1	St-15	KG-18-19	Mean	SSR 35-1	St-15	KG-18-19	Mean	SSR 35-1	St-15	KG-18-19	Mean
**OM**	**R^2^**	0.48	0.54	0.64	**0.55**	0.48	0.68	0.69	**0.62**	0.73	0.69	0.76	**0.73**
	**RMSE**	0.4	0.24	0.18	**0.27**	0.4	0.2	0.17	**0.26**	0.25	0.2	0.15	**0.20**
	**bias**	−0.03	0	0	**−0.01**	−0.03	0.01	0	**−0.01**	−0.01	0	0	**0.00**
	**RPIQ**	1.28	1.17	1.72	**1.39**	1.28	2.12	2.06	**1.82**	1.91	1.95	2.47	**2.11**
**Sand**	**R^2^**	0.41	0.5	0.55	**0.49**	0.41	0.66	0.62	**0.56**	0.57	0.59	0.73	**0.63**
	**RMSE**	8.37	9.06	6.47	**7.97**	8.37 c	7.47	6.15	**7.33**	7.01	8.41	5.22	**6.88**
	**bias**	0.14	0.3	−0.13	**0.10**	0.14	0.21	−0.08	**0.09**	0.24	−0.37	−0.21	**−0.11**
	**RPIQ**	1.46	1.25	1.47	**1.39**	1.46	2.5	1.9	**1.95**	1.95	2.45	2.37	**2.26**
**Clay**	**R^2^**	0.43	0.68	0.6	**0.57**	0.39	0.69	0.66	**0.58**	0.58	0.72	0.73	**0.68**
	**RMSE**	3.47	3.49	1.61	**2.86**	3.59	3.41	1.48	**2.83**	3.08	3.26	1.36	**2.57**
	**bias**	−0.04	0.03	0.01	**0.00**	−0.04	−0.24	0.02	**−0.09**	−0.19	−0.06	0.01	**−0.08**
	**RPIQ**	1.2	2.07	1.57	**1.61**	0.98	1.83	1.96	**1.59**	1.28	2.26	2.5	**2.01**
**Silt**	**R^2^**	0.59	0.49	0.48	**0.52**	0.54	0.55	0.55	**0.55**	0.61	0.6	0.69	**0.63**
	**RMSE**	6.16	6.62	5.7	**6.16**	6.64	6.07	5.49	**6.07**	6.12	5.85	4.53	**5.50**
	**bias**	−0.13	0.21	0.08	**0.05**	−0.03	−0.16	0.07	**−0.04**	0.1	0.17	0.18	**0.15**
	**RPIQ**	1.85	1.7	1.14	**1.56**	1.75	1.39	1.65	**1.60**	2.11	1.98	2.29	**2.13**
**B**	**R^2^**	0.67	0.62	0.25	**0.51**	0.49	0.53	0.35	**0.46**	0.81	0.7	0.49	**0.67**
	**RMSE**	0.16	1.24	0.13	**0.51**	0.22	1.4	0.12	**0.58**	0.12	1.09	0.11	**0.44**
	**bias**	0	0.07	0	**0.02**	0.01	0	0	**0.00**	0	0	0	**0.00**
	**RPIQ**	2.25	1.29	0.8	**1.45**	1.63	1.42	1.27	**1.44**	2.82	1.59	1.54	**1.98**
**Ca**	**R^2^**	0.42	0.47	0.54	**0.48**	0.32	0.5	0.55	**0.46**	0.72	0.64	0.65	**0.67**
	**RMSE**	738.46	875.83	838.52	**817.60**	776.29	857.52	832.42	**822.08**	483.76	705.47	764.21	**651.15**
	**bias**	−27.78	−21.28	−7.15	**−18.74**	11.06	−35.92	−7.16	**−10.67**	−0.97	−8.79	5.83	**−1.31**
	**RPIQ**	1.23	1.4	1.67	**1.43**	0.9	1.21	1.56	**1.22**	1.77	1.93	2.26	**1.99**
**Cu**	**R^2^**	0.24	0.45	0.45	**0.38**	0.06	0.5	0.5	**0.35**	0.74	0.53	0.56	**0.61**
	**RMSE**	0.35	1.03	0.1	**0.49**	0.36	1	0.1	**0.49**	0.19	0.95	0.09	**0.41**
	**bias**	−0.02	0.04	0	**0.01**	0	0.08	0	**0.03**	−0.01	−0.01	0	**−0.01**
	**RPIQ**	0.87	1.33	1.36	**1.19**	0.39	1.38	1.61	**1.13**	2.48	1.7	1.86	**2.01**
**Zn**	**R^2^**	0.51	0.47	0.56	**0.51**	0.42	0.41	0.64	**0.49**	0.72	0.6	0.71	**0.68**
	**RMSE**	2.59	1.43	0.72	**1.58**	2.84	1.46	0.65	**1.65**	1.86	1.23	0.59	**1.23**
	**bias**	−0.09	0.05	0	**−0.01**	−0.07	0	0	**−0.02**	−0.02	0.01	−0.01	**−0.01**
	**RPIQ**	1.02	1.34	1.54	**1.30**	0.91	1.29	1.98	**1.39**	1.23	1.73	2.03	**1.66**
**pH**	**R^2^**	0.32	0.69	0.67	**0.56**	0.6	0.74	0.77	**0.70**	0.81	0.77	0.79	**0.79**
	**RMSE**	0.23	0.6	0.5	**0.44**	0.21	0.56	0.42	**0.40**	0.12	0.53	0.4	**0.35**
	**bias**	−0.01	0.01	0	**0.00**	0.01	−0.03	0	**−0.01**	0	0	0	**0.00**
	**RPIQ**	1.18	2.11	2.41	**1.90**	1.69	2.46	2.91	**2.35**	3.37	2.45	3.01	**2.94**
